# Attitudes toward gender equality in sport among Europeans

**DOI:** 10.3389/fpsyg.2025.1529003

**Published:** 2025-03-26

**Authors:** Pamela Wicker, George B. Cunningham

**Affiliations:** ^1^Department of Sports Science, Bielefeld University, Bielefeld, Germany; ^2^Yale Divinity School, Yale University, New Haven, CT, United States

**Keywords:** female, women, media, role models, social class, sport participation

## Abstract

**Introduction:**

This study examines the role of societal, interpersonal, and individual factors in explaining Europeans’ attitudes toward gender equality in sport and the role of interactions between gender, age, and social class.

**Methods:**

The empirical analysis uses survey data from residents in 27 European countries in 2022 (*n* = 19,396). Respondents’ attitudes toward gender equality in sport are captured by two statements about evenly following female and male sport in the media and the inspirational potential of female role models among managers, athletes, officials, and coaches. Regression analyses are estimated with the attitude variables as outcomes. Societal (gender equality climate in country), interpersonal (sport participation), and individual factors (gender, age, social class; and their interactions) served as independent variables.

**Results:**

The results show that respondents have higher attitudes toward female role models than toward female sport in the media. A country’s gender equality climate has a negative association with following female sport in the media, but a positive one with female role models. Sport participation is positively associated with female sport in the media. Women score higher on both attitude measures. Older individuals and those from lower social classes evenly follow female sport in the media. Interactions between gender, age, and social class also explain attitudes toward gender equality in sport.

**Discussion:**

This study is innovative as it provides information about how the resident population thinks about gender equality in sport and which factors are relevant for higher or lower attitudes. The European-wide dataset is unique and allows a comprehensive analysis.

## 1 Introduction

Though strides have been made, gender equality in sport is not a reality. Consider, for example, that girls and women have fewer opportunities to be physically active or participate in formal sport – patterns that are evident around the world (Skauge and Seippel, 2022; Feraco et al., 2024). Furthermore, gender violence against girls and women athletes persists and limits their ability to fully participate in physical activity and sport (Nite and Nauright, 2020; Roberts et al., 2020). Gender differences are also apparent in leadership roles, where women are underrepresented as coaches, board members, and sport managers (Evans and Pfister, 2020; Hindman and Walker, 2020; Knoppers et al., 2021). Even when gains in representation are realized, traditional structures favoring men and masculinity persist (Jeanes et al., 2020).

Inequalities are also pervasive in the media coverage, marketing, and consumption of sport (Delia et al., 2022). In this case, women receive a fraction of the attention that men do, and those who do are frequently hypersexualized or come from majority backgrounds (Cooky et al., 2021; Darvin et al., 2021; Isard and Melton, 2021). The lower media coverage and media attention of female sport is problematic because especially women look for female role models (Lesch et al., 2024), but lack the possibility when male sports are prominent in the media (Wicker and Frick, 2016). Collectively, this evidence is supportive of Fink’s (2016) contention that gender inequalities are deeply embedded and taken-for-granted in sport.

The persistence of gender inequalities in sport has spurred considerable research on the topic. Much of this scholarship has focused on people directly involved in sport, including athletes, coaches, managers, and spectators (Adriaanse and Claringbould, 2014; Lagaert and Roose, 2016; de Soysa and Zipp, 2019; Harmon, 2020; Valiente, 2023). However, community members and others indirectly related to sport and physical activity hold important influence, too. These individuals shape a community’s or region’s norms and values around social topics. Organizational actors and broader industries (like sport) frequently seek to align their processes and actions with the broader community expectations, as doing so enhances legitimacy (Washington and Patterson, 2011; Nite and Edwards, 2021; Robertson et al., 2022). Furthermore, ideas about gender, equality, and sport are malleable and dependent on context and time (Wicker and Cunningham, 2023); therefore, gaining an understanding of a population’s attitudes toward gender equality and the factors that shape them is an important endeavor.

Therefore, the purpose of this study is to examine the correlates of individuals’ attitudes toward gender equality in sport. The focus is on attitudes toward consuming female sport in the media and on the inspirational potential of female role models in sport. Drawing from LaVoi’s (2016) ecological-intersectional model, we investigate the role of societal, interpersonal, and individual factors. Moreover, LaVoi (2016) also emphasizes the importance of intersectionality, recognizing that individual characteristics might interact with each other to form subsequent attitudes and perceptions. These aspects are mirrored in the two research questions of this study:

**RQ 1:** How are societal, interpersonal, and individual factors associated with individuals’ attitudes toward gender equality in sport?

**RQ 2:** How do gender, age, and social class interact to explain attitudes toward gender equality in sport?

The research context is Europe, and we utilize data from a 2022 wave of the Eurobarometer. The findings enhance our understanding of the European resident population’s attitudes toward gender equality in sport and which factors shape their perceptions.

## 2 Theoretical framework and hypotheses

We ground our work in LaVoi’s (2016) ecological-intersectional model. Like other multilevel frameworks (Cunningham, 2023), LaVoi (2016) highlighted that various factors in an ecological system – including those at the societal, organizational, interpersonal, and individual levels – are likely to influence access to sport and experiences in that context. Such a perspective is useful, as a focus on any one level of analysis will potentially tell just part of the story; instead, the ecological intersectional model makes explicit that factors at various levels of analysis shape attitudes and behaviors. As an important extension, LaVoi (2016) underscored the importance of intersectionality. That is, people’s individual characteristics are likely to intersect in synergistic ways to relate to the obstacles and facilitators they encounter (Crenshaw, 1990; Hooks, 2000). Thus, whereas women might have less access to sport than men, there is also variation among women owing to differences in social class, race, sexuality, and so on.

### 2.1 Societal factors

We considered the influence of one societal-level factor: the gender equality within the country. Consistent with the World Economic Forum (2021), a country has greater gender equality when women participate economically, are educated, are healthy, and are politically active. In such contexts, women and men have access to resources and opportunities to be successful. For example, as gender equality in a country increases, women engage in more entrepreneurial activities (Rietveld and Patel, 2022), women have greater access to finances to support their business ventures (Chundakkadan and Sasidharan, 2022), and the endorsement of gender stereotypes lessens (Wicker and Cunningham, 2023).

We suspect that these patterns might carry over to sport, too, such that people’s attitudes toward gender equality in sport mirror how women are treated, in general, in the respective country. These tenets align with research in the sport context showing that cultural values within a country are related to opportunities for women leaders and the treatment of women athletes (Ahn and Cunningham, 2017; Okada, 2023). Similarly, in their study of European Union countries, Lagaert and Roose (2016) observed that the gender equality in a country linked closely with a smaller gender gap in sport attendance. This research suggests that the gender equality in a country might influence how people perceive women in sport and their attitudes toward gender equality in sport. Thus, we hypothesized:

*H1:* A country’s gender equality climate will be positively associated with attitudes toward gender equality in sport.

### 2.2 Interpersonal factors

With respect to interpersonal factors, we focused on sport participation. Active sport participation allows for many physical and psychological benefits, but people realize social benefits, too. Consider, for example, that women constitute a sizeable share of sport club members across Europe (Nichols and Taylor, 2015; Seippel and Skille, 2015; Breuer and Feiler, 2022); thus, women and men have a chance to interact with one in various activities, such as sport competitions, social gatherings, and their participation in club operations.

The context of the interactions is also important. Sport participation is a communal experience, where people have shared goals and strive for a common outcome (Lee and Cunningham, 2014). These are the very conditions that serve to reduce intergroup anxiety and promote convivial interactions among people who are different from one another (Allport, 1954; Pettigrew and Tropp, 2006). Indeed, sport can serve as a site for reducing prejudicial norms, and it is even more effective than other bias reducing strategies, like music (Gim and Harwood, 2023).

Previous researchers have offered some support for these connections. Vezzali et al. (2022), for example, conducted a large-scale study across Europe and Asia. They found that the more people were exposed to women’s sport success, the less likely they were to endorse gender stereotypes. The patterns were especially strong for men in the study. Likewise, Wicker and Cunningham (2023) analyzed responses to the European Values Survey and observed that participation in sport clubs was linked a decrease in gender stereotypes. Collectively, these findings point to a positive association between sport participation and attitudes toward gender equality in sport. Thus, we hypothesized:

*H2:* Sport participation will be positively linked with gender equality attitudes in sport.

### 2.3 Individual factors

Finally, we considered several individual factors, including gender. Fink (2016) noted that sexism is deeply embedded in sport. Indeed, sport is an activity that was created by men and for men, and the social constructions of gender inequalities are so firmly engrained that they are institutionalized in nature (Cunningham, 2008). These dynamics are important because gender equality in sport might therefore represent a threat to the existing order or to advantages men currently enjoy. Indeed, previous researchers have noted that men’s sexist beliefs are frequently driven by their desire to maintain current societal arrangements (Sibley and Becker, 2012) and feelings of entitlement (Grubbs et al., 2014). Thus, we predicted:

*H3:* Women will express more positive sentiments about gender equality in sport than will men.

We also examined the influence of age. Previous researchers have reliably shown that younger generations hold more liberal, progressive attitudes toward social issues than do their older counterparts. Consider, for example, an extensive study from Bhatia and Bhatia (2020), who employed machine learning to examine how people thought about gender over the 20th century. The researchers showed that the strength of gender stereotypes diminished over time, largely owing to changes in how people thought about feminine characteristics. Likewise, in their study of sexism around the world, Barreto and Doyle (2023) found that, relative to their younger counterparts, older people generally expressed more biased sentiments toward women. These patterns are likely to carry over to sport, as well, especially when considering that milestone events (e.g., laws promoting girls’ and women’s sport participation; equal pay in major sport events like Wimbledon) have occurred within the past 50 years. Thus, younger people have grown up in a sport world that is more equitable than what older generations experienced. Thus, we predicted:

*H4:* Age will be negatively related to gender equality attitudes in sport.

We also considered the influence of social class. As Côté (2011) noted, social class reflects “a dimension of the self that is rooted in objective material resources (income, education and occupational prestige) and corresponding subjective perceptions of rank vis-à-vis others” (p. 47). Following this conceptualization, we considered objective and subjective measures in our treatment of social class. The influence of class on subsequent attitudes about justice and equality is mixed. On one hand, education is frequently linked with more progressive attitudes about change and people who are different from the self (Gang et al., 2013). Likewise, researchers out of Australia have shown that income holds a negative association with the endorsement of patriarchal gender beliefs (Perales and Bouma, 2018). On the other hand, social class is linked with power, status, and the subjugation of others. Results from large-scale studies suggest that subjective measures of class positively relate to systems justification, social dominance, and conservativism (Vargas-Salfate et al., 2018). The latter constructs do not correspond with support for change or greater equality for those who do not have it. Given this evidence, we predicted:

*H5:* Social class will be negatively related to gender equality attitudes in sport.

Finally, as previously noted, LaVoi (2016) emphasized the importance of intersectionality, recognizing that individual characteristics might interact with each other or with factors at other levels to predict subsequent attitudes, opportunities, and experiences. Indeed, subsequent researchers have shown that adopting an intersectional lens meaningfully contributes to the understanding of physical activity patterns (Lim et al., 2021), access to sport (Cunningham et al., 2021), sport coaching opportunities (Rankin-Wright et al., 2020), and decision making in sport organizations (Knoppers et al., 2022), among others. We explore these possibilities in our second research question.

## 3 Methods

### 3.1 Data source and sample

The study is based on data from the Eurobarometer, a regular multi-country survey of European residents which is commissioned by the European Commission and the European Parliament and conducted by Kantar Public. Specifically, the analysis uses data from the 97.3 wave which were gathered in April and May 2022. Individuals from 27 European countries (i.e., the members of the European Union) were surveyed using a multi-stage, random probability sampling procedure, ensuring that the country-specific samples are representative. The target sample size for larger countries is 1,000 respondents, while it is 500 for smaller countries such as Cyprus, Malta, and Luxembourg (European Commission and European Parliament, 2022). Overall, the raw data include *n* = 26,569 respondents. During the data cleaning process, observations with missing values on core variables were removed, leading to a complete case sample of *n* = 19,396 which can be used for the empirical analysis.

### 3.2 Variables

Table 1 provides an overview of the variables used in this study and their measurement.

**Table 1:** Overview of variables and summary statistics (*n* = 19,396).

Variable	Measurement	Mean	SD	Min	Max
Female sport media	You evenly like to follow female sport in the media (online, written, TV) as you do for male sport (1 = totally disagree, 4 = totally agree)	2.87	0.98	1	4
Female role models	Female role models among managers, athletes, officials & coaches are inspiring more women and girls to follow their example (1 = totally disagree, 4 = totally agree)	3.22	0.78	1	4
Gender equality climate	Gender gap score 2021 in country (0 = no equality, 1 = perfect equality)	0.76	0.05	0.69	0.86
PA vigorous	Number of hours per week of vigorous intensity physical activity	1.96	3.16	0	15.75
PA moderate	Number of hours per week of moderate intensity physical activity	2.29	3.37	0	15.75
PA light	Number of hours per week of light intensity physical activity	3.39	3.61	0	15.75
Female	Gender (1 = female, 0 = male)	0.524	0.499	0	1
Age	Age (in years)	49.88	17.72	15	97
Social class	Perceived social class (1 = working class of society, 5 = higher class of society)	2.52	0.96	1	5
Educational level	Respondent’s educational level (1 = very low, 32 = very high)	7.08	3.33	1	32
Not working	Employment status: Not working (1 = yes, 0 = no)	0.425	0.494	0	1
Employed	Employment status: Employed (1 = yes, 0 = no)	0.498	0.500	0	1
Self-employed	Employment status: Self-employed (1 = yes, 0 = no)	0.077	0.266	0	1
Relationship	Respondent is in a relationship (1 = yes, 0 = no)	0.643	0.479	0	1
Internet_never	Frequency of internet use: never/no access (1 = yes, 0 = no)	0.091	0.287	0	1
Internet_often	Frequency of internet use: often (1 = yes, 0 = no)	0.057	0.231	0	1
Internet_daily	Frequency of internet use: daily (1 = yes, 0 = no)	0.853	0.355	0	1
Diffpaybills_never	Difficulty paying bills last year: (almost) never (1 = yes, 0 = no)	0.666	0.472	0	1
Diffpaybills_sometimes	Difficulty paying bills last year: from time to time (1 = yes, 0 = no)	0.262	0.440	0	1
Diffpaybills_mostly	Difficulty paying bills last year: most of the time (1 = yes, 0 = no)	0.072	0.258	0	1
Life satisfaction	Satisfaction with the life you lead on the whole (1 = not at all satisfied, 4 = very satisfied)	3.08	0.68	1	4

#### 3.2.1 Attitudes toward gender equality in sport

The outcomes of interest are two variables capturing respondents’ attitudes toward gender quality in sport. The questionnaire included statements about (1) evenly following female sport in the media as for male sport (*Female sport media*) and (2) the inspirational potential of female role models among managers, athletes, officials, and coaches (*Female role models*). Respondents were asked to state their level of agreement on a four-point scale.

#### 3.2.2 Gender equality climate

At the societal level, we measured gender equality in a country (*Gender Equality*) by drawing from the global gender gap report, which is annually published by the World Economic Forum (2021). This variable is a combined score that captures women’s economic participation, educational attainment, health and survival, and political empowerment. The score ranges from *0* (no equality) to *1* (perfect equality), with various points in between. To ensure causality time-wise and given the survey data are from 2022, the 2021 gender equality measure was used.

#### 3.2.3 Sport participation

The survey contained a number of questions capturing individuals’ level of active sport participation. Respondents were asked about the weekly number of days of exercise in three different intensities (i.e., vigorous, moderate, light) and the number of minutes they spent in general for each intensity. For each intensity, the frequency variables (days) was multiplied by the duration variable (minutes per day), resulting in the number of minutes of physical activity by intensity per week. These measures were converted into hours by dividing the figure by 60, yielding the weekly number of hours for vigorous-intensity (*PA vigorous*), moderate-intensity (*PA moderate*), and light-intensity physical activity (*PA light*). Respondents who are not physically active at any of these intensities score 0 on these variables.

#### 3.2.4 Individual factors

The survey assessed a number of personal characteristics. The individual factors of interest are gender (*Female*), age (*Age*), and a subjective assessment of social class (*Social class*). The latter has five categories, from the working class of society (*1*) to the higher class of society (*5*).

#### 3.2.5 Controls

A set of individual variables was used as controls in the analysis, including educational attainment (*Education level*; is a measure from 1 to 32 which harmonizes the different education systems and degrees across Europe and makes them comparable in this combined measure), employment status (*Not working*, *Employed*, *Self-employed*), whether respondents are in some of relationship (*Relationship*), their frequency of internet use (*Internet_never; Internet_often; Internet_daily*), and their difficulty paying bills (*Diffpaybills_never; Diffpaybills_sometimes; Diffpaybills_mostly*) as a proxy for their financial situation. The study also controls for individuals’ self-reported level of life satisfaction (*Life satisfaction*).

### 3.3 Empirical analysis

The empirical analysis strategy is three-fold. First, descriptive statistics of all variables are provided to give an overview of the structure of the sample. In a second step, linear regression analyses are run to examine how societal, interpersonal, and individual factors shape the two individual attitude variables. These estimations (Models a) answer the first research question. Third, the same two regression models are estimated with interaction terms for the three individual factors of interest to answer the second research question. Specifically, gender, age, and social class are interacted with each other, and the resulting three interaction pairs are included in this second set of models (Models b). All models are linear regressions estimated using ordinary least squares, although the two attitude statements are ordinal in nature. However, existing comparative research has indicated that there are virtually no differences in findings depending on whether linear or estimators like ordered probit are employed for ordinal variables (Ferrer-i-Carbonell and Frijters, 2004).

In all models, the remaining variables from Table 1 serve as independent variables. All independent variables were checked for multicollinearity. In the first set of models excluding interaction terms, all variance inflation factors are far below the critical threshold of 10 (Hair et al., 2013), indicating that these models should not be distorted due to multicollinearity. In the second set of models with interaction terms, the variance inflation factors are slightly higher than 10 for the interaction terms and the variables used to compute these (gender, age, and social class). By construction, the variables gender, age, and social class are correlated with their interaction terms. Thus, higher VIFs are normal in models including interaction terms. For the analysis of effects, the coefficients of the normal terms and the interactions are required, which is why the normal terms should not be omitted. Typically, such regression models would control for the countries; however, including country dummies is not possible as these are highly correlated with the gender equality score. An *α*-level of 0.05 is used for determining statistical significance.

## 4 Results

Table 1 includes the summary statistics. The gender make-up shows that 52.4% of respondents are female. Average age is 49.88 years. On average, respondents tend to assign themselves to a class between lower and middle class of society (*M* = 2.52 on a five-point scale). With 7.08 on a scale from 1 to 32, respondents’ average educational level can be considered rather low. Regarding employment status, 42.7% are not working, 49.8% are in some form of employment, and 7.7% are self-employed. Altogether, 64.3% of respondents are in some form of relationship. Most respondents (85.3%) use the internet on a daily basis, 5.7% use it often (but not daily), and 9.1% never use it or have no access to the internet. Concerning respondents’ financial situation, 66.6% (almost) never have difficulty paying bills, 26.2% have such difficulty sometimes, and 7.2% have difficulty most of the time. Average life satisfaction is 3.98 with a range from 1 to 4.

Turning to the outcomes of interest, individuals have higher attitudes toward female role models in sport (*M* = 3.22 on a four-point scale) than toward evenly following female and male sport in the media (*M* = 2.87). At the societal level, the average gender equality in the 27 European countries of the study is 0.76 with a range from 0.69 to 0.86. Concerning sport participation, respondents exercise on average 1.96 h per week at vigorous intensity, 2.29 h at moderate intensity, and 3.39 h at light intensity.

Table 2 displays the regression models for the two attitude measures, with Models 1a and 2a excluding interaction terms and Models 1b and 2b including them. For models 1b and 2b, the 95% confidence intervals and the VIFs are shown as well. Gender quality climate in the country is significantly negatively associated with following female sport in the media, while having a significant positive relationship with female role models. This means that H1 can only be confirmed for female role models, while it has to be rejected for following female sport in the media. Turning to the interpersonal level, all three sport participation measures have a significant positive association with evenly following female sport in the media. They are not significant in the model for female role models. Thus, H2 is only confirmed for following female sport.

**Table 2:** Regression analyses for attitudes toward gender equality in sport (*n* = 19,396).

	Female sport media					Female role models				
	Model 1a	Model 1b	95% CI		*VIF*	Model 2a	Model 2b	95% CI		*VIF*
	*b*	*b*	*LL*	*UL*		*b*	*b*	*LL*	*UL*	
Constant	3.206***	3.384***	3.097	3.671		2.135***	2.101***	1.874	2.327	
Gender equality climate	−1.450***	−1.474***	−1.791	−1.157	1.092	0.773***	0.762***	0.513	1.012	1.092
PA vigorous	0.016***	0.017***	0.011	0.022	1.570	0.001	0.001	−0.003	0.006	1.570
PA moderate	0.005*	0.005	0.000	0.010	1.610	0.003	0.003	−0.001	0.007	1.610
PA light	0.006**	0.006**	0.002	0.010	1.201	0.003	0.003	−0.001	0.006	1.201
Female	0.098***	0.272***	0.159	0.386	17.203	0.025*	0.192***	0.103	0.282	17.203
Age	0.004***	0.003*	0.001	0.006	10.773	0.000	0.001	−0.001	0.003	10.773
Social class	−0.042***	−0.145***	−0.193	−0.098	10.999	−0.002	−0.008	−0.045	0.029	10.999
Female × Age	–	−0.006***	−0.007	−0.004	10.331	–	−0.003***	−0.004	−0.001	10.331
Female × Social class	–	0.041**	0.013	0.070	8.941	–	−0.013	−0.036	0.009	8.941
Age × Social class	–	0.002***	0.001	0.002	16.725	–	0.000	0.000	0.001	16.725
Educational level	0.014***	0.013***	0.009	0.018	1.241	0.001	0.000	−0.003	0.004	1.241
Not working	Ref.	Ref.				Ref.	Ref.			
Employed	−0.050**	−0.048**	−0.080	−0.016	1.365	−0.005	−0.004	−0.029	0.022	1.365
Self-employed	−0.069*	−0.064*	−0.119	−0.008	1.173	−0.015	−0.014	−0.058	0.030	1.173
Relationship	0.003	−0.014	−0.044	0.016	1.120	0.001	−0.007	−0.031	0.017	1.120
Internet_never	Ref.	Ref.				Ref.	Ref.			
Internet_often	0.030	0.012	−0.062	0.087	1.594	−0.102***	−0.104***	−0.163	−0.045	1.594
Internet_daily	0.239***	0.218***	0.162	0.275	2.156	0.103***	0.099***	0.054	0.144	2.156
Diffpaybills_never	Ref.	Ref.				Ref.	Ref.			
Diffpaybills_sometimes	0.012	0.009	−0.025	0.043	1.182	−0.023	−0.025	−0.051	0.002	1.182
Diffpaybills_mostly	−0.109***	−0.115***	−0.171	−0.058	1.151	0.006	0.005	−0.040	0.050	1.151
Life satisfaction	0.084***	0.084***	0.062	0.106	1.187	0.129***	0.129***	0.111	0.146	1.187
*R^2^*	0.057	0.057				0.074	0.074			
*F*	33.185***	33.185***				44.461***	44.461***			

Displayed are the unstandardized coefficients; LL = lower limit; UL = upper limit; ***p < 0.001. **p < 0.01. *p < 0.05; Ref. = reference category.

At the individual level, female gender has significant positive relationship with both attitude measures, meaning that women have higher attitudes toward gender equality in sport. These results confirm H3. Age is positively associated with following female sport in the media, while it is insignificant in the model for female role models. Thus, H4 is only partially confirmed. Perceptions of social class have a significant negative relationship with following female sport in the media, while the effect is insignificant in the model for female role models. This means that H5 is only partially supported for following female sport. Table 3 summarizes the outcomes of the hypothesis testing.

**Table 3:** Outcomes of hypothesis testing.

Hypothesis	Hypothesized effect	Female sport media	Female role models
H1 Gender equality climate in country	Positive	Not confirmed	Confirmed
H2 Sport participation	Positive	Confirmed	Not confirmed
H3 Gender (female)	Positive	Confirmed	Confirmed
H4 Age	Negative	Not confirmed	Not confirmed
H5 Social class	Negative	Confirmed	Not confirmed

The models 1b and 2b include the interaction terms. The interaction between female gender and age has a significant and negative association with both attitude measures, indicating that younger women express higher attitudes toward gender equality in sport. The interaction between female gender and social class is positive and significant in the model for following female sport in the media and insignificant in the model for female role models. Thus, women from higher social classes evenly like to follow female sport in the media. The same pattern of effects is evident for the interaction between age and social class: Older people from higher social classes like to evenly follow female and male sport in the media, while there is no significant effect for female role models.

## 5 Discussion

The purpose of this study was to examine the correlates of people’s attitudes toward gender equality in sport. We drew from the ecological intersectional model (LaVoi, 2016) to focus on factors at the societal, interpersonal, and individual levels. Results show that people generally had positive attitudes toward gender equality in sport, and that the gender equality climate in their country (societal level), their sport participation (interpersonal level), and personal characteristics (individual level) were predictive – though not always in the proposed direction and not consistent for both attitude measures. In the following space, we discuss the findings and propose some implications.

Interestingly, the gender equality climate in respondents’ country held differing associations with their attitudes toward gender equality in sport, with the effect being negative for evenly following female and male sport in the media and positive for the inspirational potential of female role models. The first finding was contrary to our expectations, as well as previous research related to gender and sport (Lagaert and Roose, 2016; Wicker and Cunningham, 2023). The latter studies focused on gender stereotypes and active sport participation in sport clubs, whereas we were interested in gender equality attitudes toward passive sport consumption in the form of watching female sport in the media. Our findings suggest that individuals living in a country with a lower level of gender equality express a greater desire to evenly follow female and male sport in the media. This means that within European countries where females are less represented in the labor force and in politics and score lower in terms of educational level and health status, individuals would like to consume as much female sport in the media as male sport.

The positive association between a country’s gender equality climate and attitudes toward female role models in sport was in line with our expectations. It echoes existing research related to gender and sport showing that a country’s level of gender equality shapes individual perceptions, attitudes, and behaviors (Lagaert and Roose, 2016; Wicker and Cunningham, 2023; Wicker et al., 2024). In the present study, the findings suggests that in countries with a more equal representation of females and males in the labor force and in political positions as well as higher education and a higher health status of females, the inspirational effect of female role models in sport for women and girls is considered higher. It is possible that in these countries respondents have learned from experience and might have witnessed inspirational effects in the labor market and in politics already. Put differently, when female representation is low in a country and only a few females hold leading positions, residents might have difficulty imagining the inspirational potential of female role models. Collectively, the findings stress the importance of the societal environment in shaping individuals’ attitudes toward gender equality in sport.

Highlighting the importance of interpersonal factors, results showed that sport participation was positively associated with attitudes toward gender equality in sport in terms of evenly following female and male sport in the media. This pattern held across all three physical activity intensities. We argue that the benefits materialize because sport is commonly a site where (a) women and men interact together, especially in Europe where many individuals practice sport in non-profit sport clubs (Nichols and Taylor, 2015; Seippel and Skille, 2015; Breuer and Feiler, 2022); (b) people can see and attest to others’ talents and contributions (Vezzali et al., 2022); and (c) and people cooperate with one another to achieve goals (Lee and Cunningham, 2014). The findings are also consistent with previous scholarship showing that the more people participate in sport clubs, the fewer gender stereotypes they endorse (Wicker and Cunningham, 2023), although the present analysis does not consider the location of physical activity.

Individual factors were also important, particularly the effects of gender and social class. Both women and people from poorer social classes expressed more positive attitudes toward gender equality than did their counterparts. These patterns likely result from issues of power and status. That is, sexism and the subjugation of women is embedded in sport (Fink, 2016), just as people from higher social classes are more likely than their peers to participate in and reap the benefits of sport (Cunningham, 2023). As gender equality represents a change to the normalized structure and pattern of arrangements, people who currently benefit the most in sport (i.e., men, older people, and people with higher social status) are unlikely to endorse changes.

The positive association between age and evenly following female and male sport in the media is contrary to expectations and existing research on gender stereotypes (Bhatia and Bhatia, 2020; Barreto and Doyle, 2023). This finding suggests that either biased sentiments toward women have decreased among older people or these sentiments do not apply to following female sport. It is possible that especially older people express a desire for a gender equal sport consumption in the media. In Europe, sport broadcasting is dominated by football (soccer), meaning that other sports including female sports are covered less. This includes public and private free-to-air networks, but also pay-tv channels. Female sport can be consumed on the internet, e.g., streams are provided on specific websites or social media websites. Model 1a documents a positive association between daily internet use and attitudes toward gender equal sport consumption in the media, supporting the importance of internet usage. However, older people tend to use the internet less for a variety of reasons.

Consistent with the ecological intersectional model (LaVoi, 2016) and theorizing related to intersectionality (Crenshaw, 1990; Hooks, 2000), personal characteristics interacted with one another to predict attitudes toward gender equality. All three interactions between female gender, age, and social class explained the even consumption of female and male sport in the media. Importantly, the effect of age turns negative at the intersection with female gender, while the effect of social class turns positive when interacted with female gender and age. This finding indicates that both female gender and age can mitigate the negative effects of social class, which tend to be driven by conservatism (Vargas-Salfate et al., 2018) and patriarchal gender beliefs (Perales and Bouma, 2018).

The importance of female role models is not shaped by social class, only by the intersection of female gender and age, with young women scoring lower on both attitude measures. It is possible that this population group is less interested in sport consumption and seeks role models from other areas than sport, such as influencers on social media. Moreover, as young women grow up in a decade with higher societal awareness for topics like gender equality and diversity, they might perceive a lower need for actively working on gender issues like gender equality in sport.

The present study makes several contributions to the body of research. Theoretically, it applies LaVoi’s (2016) ecological-intersectional model to the European population’s attitudes toward gender equality in sport. The application indicates that these attitudes are shaped by factors at the societal, interpersonal, and individual levels and that individual factors also interact with each other, supporting the usefulness of this theoretical model. Empirically, this study draws on a unique dataset covering 27 European countries allowing a comprehensive analysis. The findings provide insights not only about the level of attitudes, but also about relevant factors shaping them. Collectively, this research enhances our understanding of European’s attitudes toward different aspects of gender equality in sport and contributes to the debate about gender diversity in society and in sport.

## 6 Conclusion

This research examined the role of societal, interpersonal, and individual factors in explaining European’s attitudes toward gender equality in sport. The findings suggest that individuals’ attitudes are not only shaped by personal characteristics, but also by factors at higher levels. Different factors are at work when looking at the attitudes toward evenly following female and male sport in the media and toward the inspirational potential of female role models: With the exception of female gender, factors do not have a consistent effect on both attitude measures. Also, more factors have a significant association with following female sport in the media than with female role models. Collectively, it is not possible to develop a one-size-fits-all implication for increasing attitudes toward gender equality in sport. A nuanced look is necessary, but also future research on this topic.

Future research endeavors might also be shaped by the limitations of this work. The present research is limited by the available data and variables. This means it shares the limitations of existing studies based on cross-sectional data and it can only include measures that were assessed in the survey. For example, for a more nuanced assessment of attitudes, it would be interesting to know more about the availability of female sport in the media in each country or to each respondent. Moreover, information about whether respondents have male or female role models in sport would be insightful. Another avenue for future studies would to explore the role of other personal factors like ethnicity/race, cultural factors (e.g., religion, sporting tradition), and country-level factors like gender politics and policies within each country or the availability of women’s sport content on television and/or at streaming platforms.

## Data Availability

Publicly available datasets were analyzed in this study. This data can be found at: Doi: 10.4232/1.14055.
